# Anosognosia is associated with increased prevalence and faster development of neuropsychiatric symptoms in mild cognitive impairment

**DOI:** 10.3389/fnagi.2024.1335878

**Published:** 2024-03-06

**Authors:** Sharon Wang, Kayden Mimmack, Federica Cacciamani, Michael Elnemais Fawzy, Catherine Munro, Jennifer Gatchel, Gad A. Marshall, Geoffroy Gagliardi, Patrizia Vannini

**Affiliations:** ^1^Department of Neurology, Massachusetts General Hospital, Boston, MA, United States; ^2^Bordeaux Population Health Center, University of Bordeaux, Inserm, Bordeaux, France; ^3^Sorbonne Université, Institut du Cerveau - Paris Brain Institute - ICM, CNRS, Inria, Inserm, AP-HP, Hôpital de la Pitié Salpêtrière, Paris, France; ^4^Qarinel SAS, Paris, France; ^5^Department of Neurology, Brigham and Women's Hospital, Boston, MA, United States; ^6^Harvard Medical School, Boston, MA, United States; ^7^Department of Psychiatry, Massachusetts General Hospital, Boston, MA, United States; ^8^Division of Geriatric Psychiatry, McLean Hospital, Belmont, MA, United States

**Keywords:** awareness, Alzheimer's disease, mild cognitive impairment, neuropsychiatric symptoms, anosognosia

## Abstract

**Introduction:**

Both the loss of awareness for cognitive decline (a. k.a anosognosia) and neuropsychiatric symptoms (NPS) are common in patients with Alzheimer's disease (AD) dementia, even in prodromal stages, and may exacerbate functional impairment and negatively impact caregiver burden. Despite the high impact of these symptoms on patients and their caregivers, our knowledge of how they develop across the AD spectrum is limited. Here, we explored the cross-sectional and longitudinal associations between anosognosia and NPS in individuals with mild cognitive impairment (MCI).

**Methods:**

We included 237 participants from the Alzheimer's Disease Neuroimaging Initiative (ADNI) with a baseline clinical diagnosis of MCI. Everyday Cognition (ECog) questionnaire scores were used to measure complaints from participants and study-partners at baseline and annually over a mean of 4.29 years [standard deviation (SD) = 2.72]. Anosognosia was defined as the study-partner having an ECog score ≥2.5/4 and the participant having an ECog score < 2.5/4 on their baseline measure and their last observation without more than two consecutive deviating observations during the follow-up period. The 12-item study-partner-rated Neuropsychiatric Inventory determined the presence or absence of specific NPS. Survival analyses were performed to analyze the frequency and temporal onset of NPS over time in individuals with and without anosognosia.

**Results:**

Thirty-eight out of 237 participants displayed anosognosia. Groups had similar lengths of follow-up at baseline (*p* > 0.9), though participants with anosognosia had lower MMSE scores (*p* = 0.049) and a higher proportion of amyloid-positivity using PET (*p* < 0.001. At baseline, the frequencies of agitation (*p* = 0.029*)* and disinhibition (*p* < 0.001*)* were higher in the anosognosia group compared to the non-anosognosia group. Survival analyses showed earlier onset of seven of the 12 NPS in the anosognosia group *(p's* < 0.001).

**Discussion:**

Loss of awareness for cognitive decline is associated with greater frequency and earlier onset of NPS over time in participants with MCI. These results support the hypothesis of a potential common underlying neurophysiological process for anosognosia and NPS, a finding that needs to be addressed in future studies.

## Introduction

Alzheimer's Disease (AD) has a progressive evolution that spans several decades. During the preclinical stage, AD biomarkers are present but clinical signs or symptoms of AD are absent (Dubois et al., 2016). In mild cognitive impairment (MCI), impairment in one or more cognitive domains is seen while social or occupational functioning or functional independence are preserved (Albert et al., 2011).

Anosognosia (i.e., decreased awareness of cognitive deficits) is common at the dementia stage, estimated to be as high as 80% (Reed et al., 1993). It has also been shown to occur and develop before dementia onset (Hanseeuw et al., 2020a; Vannini et al., 2020). In addition, low self-awareness toward objective cognitive decline has been shown to predict progression from cognitively normal (CN) to MCI (Hanseeuw et al., 2020a) and from MCI to AD dementia (Gerretsen et al., 2017). Similarly, the presence and severity of anosognosia has been found to be associated with dementia progression, biological and cognitive markers of AD, and neuropsychiatric symptoms (NPS; Galeone et al., 2011; Zhao et al., 2016; Yoon et al., 2017).

Similarly, NPS are also common in the dementia stage (Zhao et al., 2016) and have been shown to be present in the pre-clinical or prodromal stages (MCI; Geda et al., 2008) as well. The most commons NPS in individuals with MCI are depression, apathy, and irritability. Meanwhile, psychotic symptoms are rare in this population (Geda et al., 2008). The presence of affective NPS, such as apathy, agitation, irritability, anxiety, and depression, are diagnostic factors that can help distinguish between CN and MCI groups, and ~50% of individuals with MCI have at least one NPS (Geda et al., 2008). Some NPS such as anxiety, hallucinations, and apathy, at baseline have been found to be associated with AD progression (Wadsworth et al., 2012).

The link between anosognosia and NPS in AD dementia is well-established. For example, a strong relationship has been observed between anosognosia and apathy in several studies (Starkstein et al., 2010; Mak et al., 2015; Azocar et al., 2021). In mild AD dementia patients, impaired awareness has been found to be associated with less depression and anxiety but greater apathy (Azocar et al., 2021). One cross-sectional study found that in mild AD dementia, anosognosia was positively correlated with agitation, aberrant motor behavior, and apathy (Spalletta et al., 2012). Another study looked at the levels of anosognosia in early-onset vs. late-onset AD dementia and its association with the presence of NPS and found that in early-onset dementia there was a strong association between early anosognosia and NPS, particularly apathy (Tondelli et al., 2021). As demonstrated above, many studies have shown that specific NPS, particularly apathy, are more common in unaware AD dementia patients.

This association has, however, not been studied as extensively in MCI. In one study, impaired awareness was found to be linked to greater severity of NPS such as euphoria, irritability, eating disorders, and aberrant motor behavior (Spalletta et al., 2012). In another study, no relationship was found between anosognosia and depression nor apathy (Mak et al., 2015).

Importantly, very few studies have looked at this relationship longitudinally. In fact, in a systematic review of the association between impaired awareness and NPS, only three of the 27 studies included were longitudinal studies (Azocar et al., 2021). One study by Starkstein et al. (2010) explored the association between anosognosia and apathy longitudinally and found that anosognosia at baseline predicts severity of apathy in the follow-up period. However, the longitudinal relationships between anosognosia and other NPS are more poorly explored (Starkstein et al., 2010). To our knowledge, Tondelli et al. (2021) is the only study that explored the relationship between anosognosia and all 12 NPS in the Neuropsychiatric Inventory-Questionnaire (NPI-Q) longitudinally (Tondelli et al., 2021). However, the association between anosognosia and the *onset* of NPS has not been explored. In addition, no study has looked at the longitudinal associations between NPS and anosognosia in participants with MCI.

Therefore, the aim of this study was to understand the association between self-awareness of memory decline and NPS at the MCI stage by investigating not only the frequency of NPS but also the onset of NPS. Given previous findings of an association between anosognosia and NPS cross-sectionally, we hypothesized that patients that are unaware of their memory deficits will show earlier onset of NPS over time.

## Materials and methods

### Population

Data used in the preparation of this article were obtained from the Alzheimer's Disease Neuroimaging Initiative (ADNI) database (adni.loni.usc.edu). ADNI is an ongoing, longitudinal, multicenter study conducted at 59 sites across North America, enrolling CN, amnestic MCI, and AD dementia participants aged 55–94 years. ADNI was launched in 2003 as a public-private partnership, led by Principal Investigator Michael W. Weiner, MD. The primary goal of ADNI has been to test whether serial magnetic resonance imaging (MRI), positron emission tomography (PET), other biological markers, and clinical and neuropsychological assessment can be combined to measure the progression of MCI and AD dementia. For up-to-date information, see www.adni-info.org.

A total of 237 ADNI participants were included in the current analyses. All participants had a baseline clinical diagnosis of MCI and longitudinal measures available for Mini-Mental State Examination (MMSE; Folstein et al., 1975), Geriatric Depression Scale (GDS; Yesavage et al., 1982) administered to the participant, Everyday Cognition (Ecog; Farias et al., 2008) scores for both participant and study partner, study partner-rated Neuropsychiatric Inventory (NPI; Cummings et al., 1994; Cummings, 1997) score, and Clinical Dementia Rating (CDR; Morris, 1993).

Demographic characteristics are summarized in Table 1.

**Table 1:** Baseline demographic measures and comparisons between participants among the anosognosia and non-anosognosia groups.

**Characteristic**	**Overall, *N* = 237**	**Anosognosia, *N* = 38**	**No anosognosia, *N* = 199**	***p*-values**
Years of observations [min; max]	4.29 ± 2.72 [0.85; 11]	4.09 ± 2.31 [0.85; 10]	4.32 ± 2.79 [0.94; 11]	>0.900
Progressors	50 (21%)	16 (42%)	34 (17%)	< 0.001
Amnestic MCI	221 (93%)	36 (95%)	185 (93%)	>0.9
Multiple domain MCI	16 (6.8%)	2 (5.3%)	14 (7.0%)	
Age (years)	73 ± 8	75 ± 7	72 ± 8	0.043
Sex (F)	105 (44%)	18 (47%)	87 (44%)	0.700
Education (years)	16.13 ± 2.77	15.97 ± 2.65	16.16 ± 2.80	0.500
MMSE (/30)	27.94 ± 1.64	27.55 ± 1.48	28.02 ± 1.66	0.049
GDS (/15)	1.78 ± 1.48	1.39 ± 1.33	1.85 ± 1.50	0.053
Amyloid load (SUVr)	1.22 ± 0.23	1.35 ± 0.23	1.19 ± 0.22	< 0.001
Amyloid group (A+)	132 (56%)	30 (79%)	102 (51%)	0.002
NPI total (/144)	3.7 ± 6.2	6.7 ± 7.4	3.1 ± 5.8	< 0.001

MMSE, Mini-Mental State Examination; GDS, Geriatric Depression Scale; NPI, Neuropsychiatric Inventory; SUVr, Standard Uptake Value ratio.

Means ± standard deviations are included for continuous variables and count and percentages are included for categorical variables.

### Awareness measure

ECog (Farias et al., 2008) questionnaire scores were used to measure complaints from participants and study partners over the course of the study. The ECog scale includes six subscales (i.e., Memory, Language, Visual-spatial/Perceptual Abilities, Planning, Organization, and Divided-Attention) exploring cognitive domains over 39 questions. For each question, the respondent is asked to compare the current level of cognitive functioning to 10 years ago using concrete examples (e.g., “*Remembering a few shopping items without a list*”). The rating is done on a Likert scale from 1 (“Better or no change”) to 4 (“Consistently much worse”), with a higher score describing the perception of a greater cognitive decline. Consistent with prior literature in MCI and AD dementia samples (Hanseeuw et al., 2020b; Gagliardi et al., 2021), we focused on the eight questions of the memory subscale. We computed the participant and study partner average scores and defined the presence or absence of anosognosia depending on these values. Participants were considered to have low awareness of cognitive decline when their average complaint score was < 2.5/4 while their study partner's rating was ≥2.5/4. The rationale for this approach was to create two groups in which the participant and study partner were on opposite sides of the ECog scale. This grouping method was used for the baseline observation and all longitudinal observations and, similarly to the method previously used by Starkstein et al. (2010), we extracted participants showing stable anosognosia as opposed to the consistent absence of anosognosia. Therefore, at least two ECog assessments were needed to determine stable anosognosia. Stability was defined as a similar grouping from baseline to the last observation, without having more than two consecutive “deviating” observations during the follow-up period. For example, a participant who displayed anosognosia at baseline and their second follow-up but not at their first follow-up would be characterized as someone with stable anosognosia.

### Neuropsychiatric symptoms

The presence of NPS was assessed using the Neuropsychiatric Inventory (NPI; Cummings et al., 1994; Cummings, 1997). The NPI is a study partner-rated questionnaire assessing the presence and severity/frequency of specific NPS. Twelve domains are explored: delusions, hallucinations, agitation/aggression, depression/dysphoria, anxiety, elation/euphoria, apathy/indifference, disinhibition, irritability/lability, aberrant motor behavior, sleep, and appetite and eating disorders. For each of these categories, the study partner is first asked whether the described symptom is observed in the participant. If the symptom is observed, study partners are then asked about the severity (i.e., 1 = Mild, 2 = Moderate, and 3 = Severe) and the frequency (1 = rarely, less than once per week; 2 = sometimes, about once per week; 3 = often, several times per week; and 4 = very often, once, or more per day) of the symptom. If the symptom is not observed, these questions are skipped. The NPI total score is computed by summing each of the domain scores, which is calculated by multiplying the severity and frequency scores (a maximum score of 12 per domain), and the NPI total has a maximum score of 144 with higher scores indicating greater symptoms.

### Imaging

All participants underwent Florbetapir (^18^*F*−*AV*45) PET, used to measure brain amyloidosis. We used a whole brain standard uptake value ratio (SUVr) in large areas of the frontal, lateral temporal, and parietal lobes, based on the whole cerebellum as a reference region. For group comparisons, participants were also classified as amyloid positive (A+) or negative (A–) using a 1.11 cut-off as previously described for the ADNI cohort (Landau et al., 2013).

### Statistical analyses

We first compared baseline observations of participants with and without stable anosognosia. Continuous variables, including age and education (in years), MMSE and NPI total scores (/30 and/144, respectively), brain amyloidosis (using the global SUVr at PET) and the number of observations, were compared using Wilcoxon rank sum test. As for categorical variables such as sex (F/M) and amyloid grouping (A+/A-), we used Pearson's Chi-squared test.

Survival analyses were performed to analyze the rate of appearance of NPS over time in individuals with and without anosognosia. We applied Cox proportional hazards regression models, using the presence or absence of a determined NPI as the predicted value, and the awareness grouping (i.e., presence or absence of anosognosia) as the predictor of interest. Demographics (i.e., age, sex and education) and baseline MMSE were added as covariates, and estimate log hazard ratios (HRs) were computed with 95% confidence intervals (CI). Separate models adding amyloidosis and baseline values for baseline phosphorylated tau cerebrospinal fluid as covariates were run as well. Kaplan–Meier curves were computed to visualize the results. *P*-values were corrected for multiple comparisons using the Bonferroni (1939) method. All statistical analyses were performed using R4.1.0 (https://www.R-project.org/). The survival (Therneau and Grambsch, 2000; Therneau, 2023) and survminer (Kassambara et al., 2021) packages were used to compute the survival analyses and their graphic representations.

## Results

### Baseline comparisons

Following the longitudinal grouping criteria we described, we split our sample into two groups of participants depending on whether they showed a stable presence or absence of anosognosia. Eighty-four percent of our sample, i.e., 199 participants out of 237, met our criteria for the absence of anosognosia. The remaining 16%, i.e., 38 participants, were considered to have anosognosia.

As reported in Table 1, participants with and without anosognosia showed similar baseline demographic characteristics (i.e., sex and years of education) and years of observations. Participants with anosognosia were older and had lower MMSE scores. Comparing brain amyloidosis levels, the anosognosia group showed greater amyloidosis and a corresponding greater proportion of amyloid-positive participants. Additionally, the anosognosia group was more likely to be progressors, demonstrating a clinical progression from MCI to AD dementia at some point in the follow-up period. Finally, the group with anosognosia showed more NPS at baseline as compared to the participants without anosognosia with a greater NPI Total score. However, the group with anosognosia and the group without anosognosia had similar GDS scores.

We also compared these two groups at baseline on the frequency of NPS. As reported in Table 2, participants with anosognosia showed a greater frequency of NPS on agitation and disinhibition compared to participants without anosognosia.

**Table 2:** Baseline comparisons of frequency of NPS between participants among the anosognosia and non-anosognosia groups.

**NPS**	**Overall, *N* = 237**	**Anosognosia, *N* = 38**	**No anosognosia, *N* = 199**	***p*-values**
Delusions	3 (1.3%)	0 (0%)	3 (1.5%)	>0.900
Hallucinations	3 (1.3%)	1 (2.6%)	2 (1.0%)	0.400
Agitation/aggression	35 (15%)	10 (26%)	25 (13%)	0.029
Depression/dysphoria	63 (27%)	11 (29%)	52 (26%)	0.700
Anxiety	29 (12%)	7 (18%)	22 (11%)	0.300
Elation/euphoria	3 (1.3%)	0 (0%)	3 (1.5%)	>0.900
Apathy/indifference	34 (14%)	9 (24%)	25 (13%)	0.073
Disinhibition	19 (8.0%)	10 (26%)	9 (4.5%)	< 0.001
Irritability/lability	50 (21%)	12 (32%)	38 (19%)	0.084
Aberrant motor behavior	9 (3.8%)	3 (7.9%)	6 (3.0%)	0.200
Sleep disorders	51 (22%)	12 (32%)	39 (20%)	0.100
Appetite and eating disorders	18 (7.6%)	4 (11%)	14 (7.0%)	0.500

### Survival analyses

**Figure 1:**
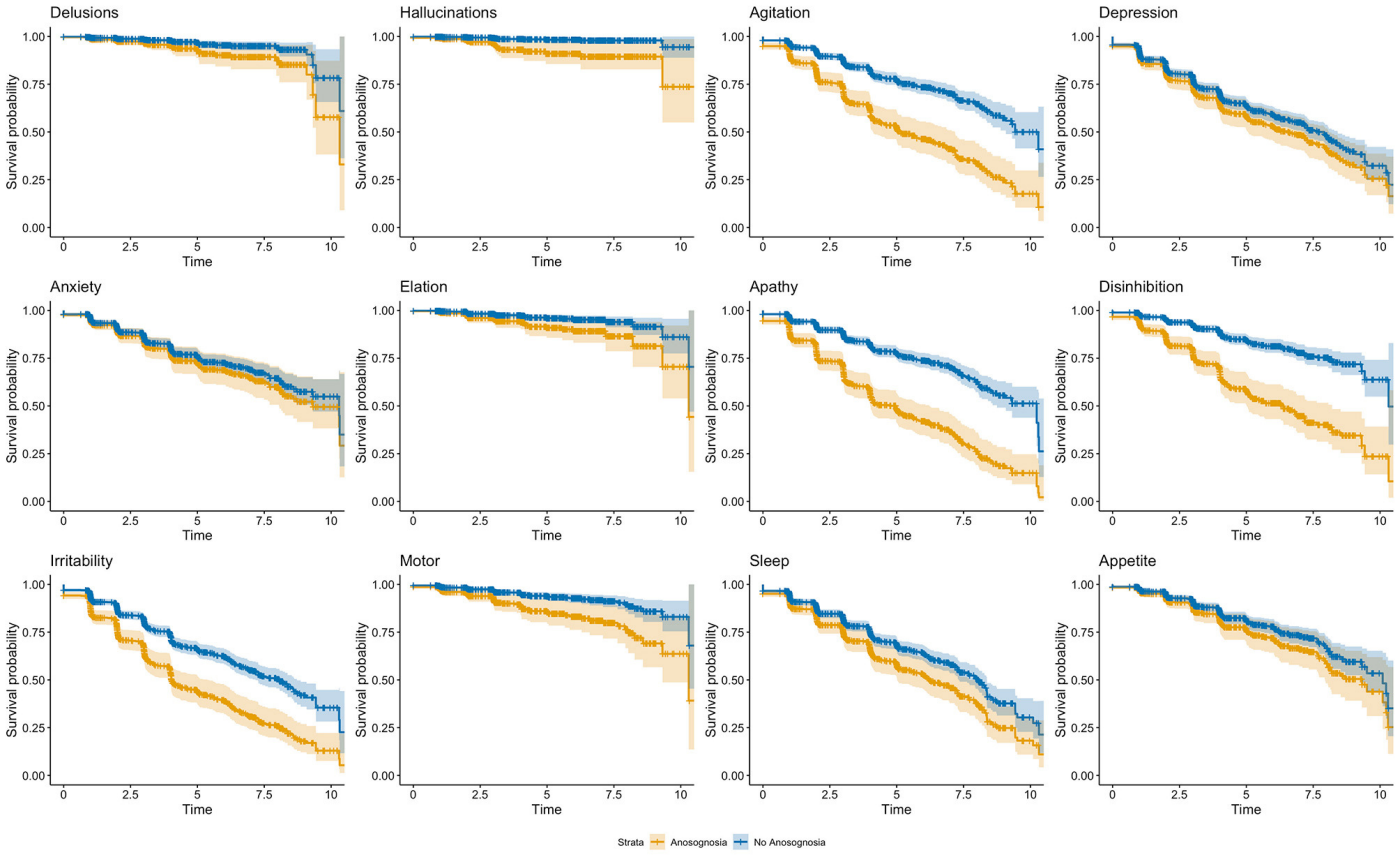
Cox regression model outputs comparing the onset of NPS over time for participants with and without stable anosognosia. The orange line indicates participants with anosognosia, and the blue line indicates participants without anosognosia.

After Bonferroni correction for multiple comparisons, no significant effects of age nor education were observed within the different models (see Table 3). There was a significant effect of sex on the onset of agitation, elation, and irritability so that males had smaller risks of developing agitation and irritability and had greater risks of developing elation. Additionally, lower baseline MMSE were significantly related to greater risk of progression in anxiety, apathy, and appetite and eating disorders. Regarding the groups with and without anosognosia, a significant difference was observed for seven of the 12 NPI domains (delusions, hallucinations, agitation, apathy, disinhibition, irritability, and aberrant motor behavior) such that participants with anosognosia showed an earlier onset of NPS over time as compared to participants without anosognosia (see Table 3 and Figure 1).

**Table 3:** Cox regression model outputs comparing the onset of NPS over time for participants with and without stable anosognosia.

**NPS**	**Age (baseline)**	**Sex (M)**	**Education (years)**	**Baseline MMSE**	**Group (anosognosia)**
**Delusions**					
log (HR)	−0.03	−0.72	0.07	−0.22	1.40
95% CI	−0.08, 0.03	−1.50, 0.09	−0.06, 0.20	−0.49, 0.04	0.60, 2.20
*p*-value	1.000	0.982	1.000	1.000	0.007
**Hallucinations**					
log(HR)	−0.05	−1.00	−0.04	0.01	2.10
95% CI	−0.13, 0.03	−2.20, 0.10	−0.23, 0.15	−0.44, 0.46	0.97, 3.20
*p*-value	1.000	0.896	1.000	1.000	0.003
**Agitation/aggression**					
log(HR)	−0.01	0.56	−0.01	−0.12	0.73
95% CI	−0.03, 0.01	0.22, 0.91	−0.06, 0.05	−0.22, −0.02	0.40, 1.00
*p*-value	1.000	0.017	1.000	0.186	< 0.001
**Depression/dysphoria**					
log(HR)	0.01	−0.11	0.02	−0.03	0.30
95% CI	−0.01, 0.03	−0.41, 0.19	−0.03, 0.07	−0.13, 0.06	−0.05, 0.65
*p*-value	1.000	1.000	1.000	1.000	1.000
**Anxiety**					
log(HR)	−0.01	−0.14	0.04	−0.19	−0.09
95% CI	−0.04, 0.01	−0.48, 0.19	−0.02, 0.09	−0.29, −0.09	−0.54, 0.37
*p*-value	1.000	1.000	1.000	0.003	1.000
**Elation/euphoria**					
log(HR)	−0.03	1.80	−0.15	0.43	0.78
95% CI	−0.08, 0.02	0.76, 2.90	−0.27, −0.03	0.07, 0.79	0.04, 1.50
*p*-value	1.000	0.010	0.165	0.240	0.469
**Apathy/indifference**					
log(HR)	0.02	−0.33	0.04	−0.20	1.10
95% CI	0.00, 0.04	−0.64, −0.03	−0.02, 0.09	−0.29, −0.11	0.81, 1.40
*p*-value	0.763	0.401	1.000	< 0.001	< 0.001
**Disinhibition**					
log(HR)	−0.01	0.31	−0.01	−0.01	0.89
95% CI	−0.04, 0.02	−0.09, 0.71	−0.07, 0.06	−0.14, 0.13	0.50, 1.30
*p*-value	1.000	1.000	1.000	1.000	< 0.001
**Irritability/lability**					
log(HR)	−0.01	0.58	0.01	−0.11	0.66
95% CI	−0.03, 0.01	0.29, 0.87	−0.03, 0.05	−0.19, −0.03	0.38, 0.93
*p*-value	1.000	< 0.001	1.000	0.091	< 0.001
**Aberrant motor behavior**					
log(HR)	0.02	−0.10	−0.05	0.10	0.97
95% CI	−0.02, 0.06	−0.68, 0.48	−0.14, 0.05	−0.12, 0.31	0.36, 1.60
*p*-value	1.000	1.000	1.000	1.000	0.022
**Sleep disorders**					
log(HR)	−0.02	0.43	0.03	−0.10	−0.09
95% CI	−0.04, 0.00	0.11, 0.75	−0.02, 0.08	−0.19, −0.01	−0.47, 0.29
*p*-value	1.000	0.094	1.000	0.399	1.000
**Appetite and eating disorders**					
log(HR)	0.01	−0.52	0.07	−0.18	0.23
95% CI	−0.01, 0.03	−0.87, −0.16	0.00, 0.13	−0.28, −0.07	−0.20, 0.66
*p*-value	1.000	0.050	0.432	0.010	1.000

P-values are corrected for multiple comparisons using the Bonferroni method.

The results of the model adding amyloidosis as a covariate are presented in Supplementary Table 1. The results of the model adding amyloidosis and baseline CSF phosphorylated tau as covariates are presented in Supplementary Table 2.

## Discussion

In this study, we investigated the association between anosognosia and the frequency and development of NPS in individuals with MCI. We observed a significantly earlier onset of seven of the 12 NPS in participants with anosognosia as compared to participants without anosognosia. These seven NPS include delusion, hallucination, agitation, apathy, disinhibition, irritability, and aberrant motor behavior. This study adds to the existing literature by exploring the time to development of NPS using the presence or absence of anosognosia as a predictor. In addition, we assessed baseline comparisons between the two groups on some cognitive, neuropsychiatric, and pathological variables. Our results showed that at baseline, participants classified as having anosognosia demonstrated lower MMSE scores and greater cerebral amyloid levels, in line with previous findings (Hanseeuw et al., 2020b; Gagliardi and Vannini, 2022) indicating that anosognosia is related to AD pathology. Participants with anosognosia had more NPS at baseline than those without, which is consistent with previous results in the dementia population (Spalletta et al., 2012; Conde-Sala et al., 2016; Martyr et al., 2022). Specifically, agitation and disinhibition were more frequent in MCI participants with anosognosia compared to those without (see Table 2). These results are in line with Spalletta et al. (2012) who found that in individuals with amnestic multidomain MCI, impaired awareness was related to greater severity of disinhibition. Yoon et al. (2017) also found that in participants with CDR scores of 0.5, the prevalence of agitation was higher in those with anosognosia compared to those without. Studies that have looked at cross-sectional associations between anosognosia and NPS in dementia have found positive relationships between anosognosia and behavioral disturbances like agitation and disinhibition (Aalten et al., 2005; Kashiwa et al., 2005; Spalletta et al., 2012).

In our sample, depression was not associated with anosognosia at baseline using the depression item in the NPI-Q nor the self-reported GDS. Some studies have also found no association between depression and anosognosia, which may reflect differences in depressive symptoms from dysthymia or mild depression vs. major depression (Aalten et al., 2005).

Whereas, previous researchers have consistently found apathy and anosognosia to be positively related in the dementia population (Aalten et al., 2005; Starkstein et al., 2010; Tondelli et al., 2021), we did not find that participants with anosognosia had more apathy at baseline. Our results agree with those of Mak et al. (2015) which did not find any association between apathy and anosognosia in MCI but did in their participants with AD dementia. This could be explained by the fact that participants with MCI demonstrate a lower prevalence of NPS than in AD dementia (Geda et al., 2008).

Our longitudinal study aim was to understand the association between anosognosia and the potential development of NPS while adjusting for baseline age, sex, years of education, and MMSE. To our knowledge, this study is the first longitudinal study to investigate the association between anosognosia and the onset of NPS in MCI participants. Our findings are consistent with previous studies in AD dementia that have found anosognosia at baseline to be associated with the development of future NPS, in particular apathy. Starkstein et al. (2010) and Tondelli et al. (2021) found that in the dementia population, anosognosia at baseline predicted more severe apathy at follow-up and that higher levels of anosognosia were associated with subsequent apathy, respectively. However, it should be noted that those studies were done in the dementia population only.

Our longitudinal results, in combination with the cross-sectional findings, suggest that there is an association between anosognosia and NPS. Indeed, previous neuroimaging studies have found that both apathy and anosognosia significantly correlate with right hemisphere dysfunction, specifically the frontal regions, in AD dementia (Ott et al., 1996). In addition, changes to the anterior cingulate cortex is common across almost all NPS (Chen et al., 2021) and are also associated with anosognosia (Valera-Bermejo et al., 2020). Decreased connectivity in the default mode network (DMN), a brain region that supports self-referential processing, has also been linked to both anosognosia (Antoine et al., 2019) and NPS (Lee et al., 2020), specifically hyperactivity symptoms such as agitation, irritability, aberrant motor behavior, euphoria, and disinhibition. While there may not be a direct causal relationship between the two, it is possible that their co-occurrence could be explained by shared neurobiological mechanisms in the frontal lobe, including in the orbitofrontal, prefrontal, and anterior cingulate cortex (Aalten et al., 2005; Starkstein et al., 2010; Spalletta et al., 2012). Therefore, awareness and NPS may both depend on the functioning of the same neural substrates. Interestingly, in our study, participants with anosognosia demonstrated greater global cerebral amyloid levels. This finding is in accordance with previous findings (Vannini et al., 2017; Cacciamani et al., 2020; Hanseeuw et al., 2020b; Gagliardi et al., 2021). Future studies are needed to understand if increased AD pathology might contribute to the joint dysfunction of awareness and NPS.

### Limitations

We acknowledge that this study has some limitations. First, we acknowledge that there are a variety of methods to assess anosognosia and NPS. We assessed the prevalence of NPS with study partner-rated NPI scores and the presence of anosognosia by calculating the discrepancy between study partner and participant E-Cog questionnaire scores. However, there is a possibility that study partners may be hypervigilant and over-report NPS and difficulties with everyday abilities. Similarly, anosognosia was defined as the study-partner having an ECog score ≥2.5/4 and the participant having an ECog score < 2.5/4 on their baseline measure and their last observation without more than two consecutive deviating observations during the follow-up period. We acknowledge that a different definition of stable anosognosia may yield different results. Additionally, study partners may report affective changes (i.e., how the participant is presenting outwardly to others), and their score may not accurately reflect the participant's internalized symptoms. Other alternative assessments that could have been used include clinician-rated evaluations or participant-rated questionnaires (i.e., for awareness, a discrepancy between participant's subjective and objective performances). Future studies are need to replicate our findings using these alternative approaches. Next, this cohort was a highly educated sample that may not be representative of the general population, and future studies should investigate this association using a more heterogeneous sample. Finally, we acknowledge the limited follow-up duration. Future studies should look at this association with longer follow-up to confirm our findings.

## Conclusion

In conclusion, our findings provide further support for an association between anosognosia and NPS in individuals diagnosed with MCI at baseline. Participants who were unaware of their memory decline displayed more NPS at baseline and developed NPS earlier than participants who were aware of their cognitive decline. Individuals with anosognosia also had higher frequency of clinical decline over time. Subsequently, these findings suggest that individuals with MCI and anosognosia may be at greater risk for developing NPS and AD dementia. This information is important to relay to clinicians as it may help in the development of clinical interventions that could lessen the combined negative impact of anosognosia and NPS on patients' wellbeing. The findings from this study also support the hypothesis of a potential common underlying neurophysiological process for these manifestations. Future studies should investigate this association using neuroimaging techniques.

## Data availability statement

Publicly available datasets were analyzed in this study. Data used in the preparation of this article were obtained from the Alzheimer's Disease Neuroimaging Initiative (ADNI) database (adni.loni.usc.edu).

## Author contributions

SW: Formal analysis, Investigation, Visualization, Writing – original draft. KM: Writing – review & editing. FC: Writing – review & editing. ME: Writing – review & editing. CM: Writing – review & editing. JG: Writing – review & editing. GM: Writing – review & editing. GG: Formal analysis, Investigation, Supervision, Visualization, Writing – original draft. PV: Conceptualization, Investigation, Methodology, Resources, Supervision, Writing – review & editing.
