# Supplementary material for: Anosognosia is associated with increased prevalence and faster development of neuropsychiatric symptoms in mild cognitive impairment
Source: Front Aging Neurosci. 2024 Mar 6;16:1335878. doi: 10.3389/fnagi.2024.1335878 (PMC10950916; doi:10.3389/fnagi.2024.1335878)
Supplement: Supplementary file 1 [file Data_Sheet_1.docx]

Supplementary Material

Anosognosia is associated with increased prevalence and faster development of neuropsychiatric symptoms in mild cognitive impairment

Wang, S.^1^, Mimmack, K.^1^, Cacciamani, F.^1,2,5,6^, Elnemais, M.^3,4^, Munro, C.^1,3,4^, Gatchel, J.^4,7,8^, Marshall, G.^1,3,4^, Gagliardi, G. †^1,3,4^, Vannini, P. †*^1,3,4^, for the Alzheimer’s Disease Neuroimaging Initiative

†These authors share senior authorship.

^1^Department of Neurology, Massachusetts General Hospital, Boston, MA, USA

^2^Bordeaux Population Health Center, University of Bordeaux, Inserm, Bordeaux, France

^3^Department of Neurology, Brigham and Women’s Hospital, Boston, MA, USA

^4^Harvard Medical School, Boston, MA, USA

^5^Sorbonne Université, Institut du Cerveau - Paris Brain Institute - ICM, CNRS, Inria, Inserm, AP-HP, Hôpital de la Pitié Salpêtrière, Paris, France

^6^Qarinel SAS, Paris, France

^7^Department of Psychiatry, Massachusetts General Hospital, Boston, MA, USA

^8^Division of Geriatric Psychiatry, McLean Hospital, Belmont, MA, USA

*** Correspondence:**Patrizia Vannini
patrizia@bwh.harvard.edu

# Supplementary Method

## *CSF Ptau*

CSF pTau data (UPENNBIOMK10_07_29_19.csv) were downloaded from ADNI, using the analyses performed by the University of Pennsylvania ADNI Biomarker core laboratory. These CSF values were based on the Roche Elecsys and cobas e 601 immunoassay analyzer system. Among the 237 participants in the selected sample, 215 presented available CSF-PTAU measures (37 in the Anosognosia group and 178 in the Non-Anosognosia group).

# Supplementary Tables

**Supplementary Table 1.** Cox regression model outputs comparing the onset of NPS over time for participants with and without stable anosognosia. Covariates include those in the original analysis as well as amyloidosis. P-values are corrected for multiple comparisons using the Bonferroni method.

| NPS | Age (Baseline) | Sex (M) | Education (years) | Baseline MMSE | Amyloidosis | Group (**Anosognosia**) |
| --- | --- | --- | --- | --- | --- | --- |
| Delusions |  |  |  |  |  |  |
| log(HR) | -0.02 | -0.54 | 0.07 | -0.18 | 3.70 | 1.00 |
| 95% CI | -0.09, 0.04 | -1.30, 0.23 | -0.08, 0.22 | -0.46, 0.10 | 2.10, 5.30 | 0.25, 1.80 |
| p-value | 1.000 | 1.000 | 1.000 | 1.000 | <0.001 | 0.117 |
| Hallucinations |  |  |  |  |  |  |
| log(HR) | -0.06 | -0.71 | -0.05 | 0.06 | 3.10 | 1.70 |
| 95% CI | -0.15, 0.03 | -1.80, 0.37 | -0.26, 0.16 | -0.40, 0.52 | 0.84, 5.40 | 0.57, 2.80 |
| p-value | 1.000 | 1.000 | 1.000 | 1.000 | 0.091 | 0.037 |
| Agitation / Aggression |  |  |  |  |  |  |
| log(HR) | -0.01 | 0.60 | 0.00 | -0.11 | 1.30 | 0.65 |
| 95% CI | -0.03, 0.01 | 0.26, 0.95 | -0.05, 0.05 | -0.21, -0.01 | 0.65, 1.90 | 0.33, 0.98 |
| p-value | 1.000 | 0.006 | 1.000 | 0.334 | <0.001 | <0.001 |
| Depression / Dysphoria |  |  |  |  |  |  |
| log(HR) | 0.01 | -0.11 | 0.02 | -0.03 | 0.61 | 0.26 |
| 95% CI | -0.01, 0.03 | -0.41, 0.18 | -0.03, 0.07 | -0.12, 0.07 | -0.02, 1.20 | -0.09, 0.61 |
| p-value | 1.000 | 1.000 | 1.000 | 1.000 | 0.716 | 1.000 |
| Anxiety |  |  |  |  |  |  |
| log(HR) | -0.01 | -0.13 | 0.03 | -0.18 | 0.91 | -0.15 |
| 95% CI | -0.03, 0.01 | -0.46, 0.20 | -0.03, 0.09 | -0.28, -0.08 | 0.21, 1.60 | -0.61, 0.30 |
| p-value | 1.000 | 1.000 | 1.000 | 0.004 | 0.129 | 1.000 |
| Elation / Euphoria |  |  |  |  |  |  |
| log(HR) | -0.03 | 1.80 | -0.15 | 0.43 | 0.00 | 0.78 |
| 95% CI | -0.08, 0.02 | 0.76, 2.90 | -0.27, -0.03 | 0.07, 0.79 | -1.70, 1.70 | 0.04, 1.50 |
| p-value | 1.000 | 0.010 | 0.165 | 0.241 | 1.000 | 0.472 |
| Apathy / Indifference |  |  |  |  |  |  |
| log(HR) | 0.02 | -0.24 | 0.03 | -0.18 | 1.40 | 0.99 |
| 95% CI | 0.00, 0.04 | -0.55, 0.06 | -0.02, 0.09 | -0.27, -0.09 | 0.76, 2.10 | 0.68, 1.30 |
| p-value | 0.866 | 1.000 | 1.000 | <0.001 | <0.001 | <0.001 |
| Disinhibition |  |  |  |  |  |  |
| log(HR) | -0.01 | 0.33 | -0.01 | 0.01 | 1.40 | 0.82 |
| 95% CI | -0.04, 0.02 | -0.07, 0.72 | -0.07, 0.06 | -0.12, 0.14 | 0.66, 2.20 | 0.44, 1.20 |
| p-value | 1.000 | 1.000 | 1.000 | 1.000 | 0.004 | <0.001 |
| Irritability / Lability |  |  |  |  |  |  |
| log(HR) | -0.01 | 0.61 | 0.01 | -0.10 | 0.91 | 0.60 |
| 95% CI | -0.02, 0.01 | 0.32, 0.89 | -0.03, 0.06 | -0.18, -0.02 | 0.31, 1.50 | 0.33, 0.88 |
| p-value | 1.000 | <0.001 | 1.000 | 0.210 | 0.034 | <0.001 |
| Aberrant Motor Behavior |  |  |  |  |  |  |
| log(HR) | 0.02 | -0.04 | -0.06 | 0.13 | 3.0 | 0.74 |
| 95% CI | -0.02, 0.06 | -0.61, 0.53 | -0.16, 0.05 | -0.10, 0.35 | 1.90, 4.10 | 0.13, 1.30 |
| p-value | 1.000 | 1.000 | 1.000 | 1.000 | <0.001 | 0.205 |
| Sleep Disorders |  |  |  |  |  |  |
| log(HR) | -0.02 | 0.43 | 0.03 | -0.10 | 0.30 | -0.10 |
| 95% CI | -0.04, 0.00 | 0.12, 0.75 | -0.02, 0.08 | -0.19, -0.01 | -0.39, 0.98 | -0.48, 0.28 |
| p-value | 1.000 | 0.087 | 1.000 | 0.447 | 1.000 | 1.000 |
| Appetite and Eating Disorders |  |  |  |  |  |  |
| log(HR) | 0.01 | -0.48 | 0.07 | -0.17 | 1.30 | 0.11 |
| 95% CI | -0.01, 0.03 | -0.83, -0.13 | 0.00, 0.13 | -0.28, -0.07 | 0.55, 2.00 | -0.33, 0.54 |
| p-value | 1.000 | 0.089 | 0.529 | 0.015 | 0.007 | 1.000 |

**Supplementary Table 2.** Cox regression model outputs comparing the onset of NPS over time for participants with and without stable anosognosia. Covariates include those in the original analysis as well as amyloidosis and CSF pTau. P-values are corrected for multiple comparisons using the Bonferroni method.

| NPS | Age (Baseline) | Sex (M) | Education (years) | Baseline MMSE | Amyloidosis | CSF pTau | Group (Anosognosia) |
| --- | --- | --- | --- | --- | --- | --- | --- |
| Delusions |  |  |  |  |  |  |  |
| log(HR) | -0.07 | -1.10 | 0.13 | -0.16 | 4.30 | -0.01 | 1.00 |
| 95% CI | -0.14, 0.00 | -1.90, -0.16 | -0.04, 0.29 | -0.48, 0.17 | 2.40, 6.10 | -0.03, 0.01 | 0.18, 1.90 |
| p-value | 0.750 | 0.248 | 1.000 | 1.000 | <0.001 | 1.000 | 0.211 |
| Hallucinations |  |  |  |  |  |  |  |
| log(HR) | -0.17 | -1.50 | 0.04 | 0.43 | 5.40 | -0.03 | 2.50 |
| 95% CI | -0.29, -0.05 | -2.80, -0.17 | -0.20, 0.29 | -0.34, 1.20 | 2.30, 8.60 | -0.07, 0.01 | 0.93, 4.00 |
| p-value | 0.081 | 0.316 | 1.000 | 1.000 | 0.008 | 1.000 | 0.020 |
| Agitation / Aggression |  |  |  |  |  |  |  |
| log(HR) | 0.00 | 0.48 | -0.02 | -0.09 | 1.40 | 0.00 | 0.64 |
| 95% CI | -0.02, 0.02 | 0.12, 0.83 | -0.08, 0.04 | -0.20, 0.01 | 0.65, 2.10 | 0.00, 0.01 | 0.30, 0.97 |
| p-value | 1.000 | 0.103 | 1.000 | 0.981 | 0.002 | 1.000 | 0.002 |
| Depression / Dysphoria |  |  |  |  |  |  |  |
| log(HR) | 0.00 | -0.29 | 0.02 | 0.00 | 0.62 | 0.00 | 0.28 |
| 95% CI | -0.02, 0.02 | -0.61, 0.02 | -0.03, 0.08 | -0.11, 0.10 | -0.13, 1.40 | 0.00, 0.01 | -0.08, 0.63 |
| p-value | 1.000 | 0.794 | 1.000 | 1.000 | 1.000 | 1.000 | 1.000 |
| Anxiety |  |  |  |  |  |  |  |
| log(HR) | -0.01 | -0.11 | 0.03 | -0.18 | 0.80 | 0.00 | -0.19 |
| 95% CI | -0.04, 0.01 | -0.47, 0.25 | -0.03, 0.09 | -0.29, -0.08 | -0.01, 1.60 | -0.01, 0.01 | -0.65, 0.28 |
| p-value | 1.000 | 1.000 | 1.000 | 0.011 | 0.635 | 1.000 | 1.000 |
| Elation / Euphoria |  |  |  |  |  |  |  |
| log(HR) | -0.05 | 1.40 | -0.13 | 0.32 | 0.46 | -0.01 | 0.68 |
| 95% CI | -0.10, 0.01 | 0.33, 2.50 | -0.26, 0.00 | -0.07, 0.71 | -1.50, 2.50 | -0.03, 0.01 | -0.11, 1.50 |
| p-value | 1.000 | 0.128 | 0.624 | 1.000 | 1.000 | 1.000 | 1.000 |
| Apathy / Indifference |  |  |  |  |  |  |  |
| log(HR) | 0.02 | -0.34 | 0.04 | -0.19 | 1.60 | 0.00 | 0.92 |
| 95% CI | -0.01, 0.04 | -0.66, -0.02 | -0.01, 0.10 | -0.29, -0.09 | 0.82, 2.40 | -0.01, 0.01 | 0.60, 1.20 |
| p-value | 1.000 | 0.447 | 1.000 | 0.002 | <0.001 | 1.000 | <0.001 |
| Disinhibition |  |  |  |  |  |  |  |
| log(HR) | -0.02 | 0.15 | 0.00 | -0.01 | 1.70 | -0.01 | 0.71 |
| 95% CI | -0.05, 0.01 | -0.27, 0.56 | -0.07, 0.06 | -0.15, 0.13 | 0.84, 2.50 | -0.02, 0.00 | 0.32, 1.10 |
| p-value | 1.000 | 1.000 | 1.000 | 1.000 | <0.001 | 0.552 | 0.004 |
| Irritability / Lability |  |  |  |  |  |  |  |
| log(HR) | 0.00 | 0.47 | 0.01 | -0.08 | 0.89 | 0.00 | 0.57 |
| 95% CI | -0.02, 0.01 | 0.18, 0.77 | -0.04, 0.06 | -0.17, 0.00 | 0.21, 1.60 | -0.01, 0.01 | 0.28, 0.85 |
| p-value | 1.000 | 0.022 | 1.000 | 0.726 | 0.128 | 1.000 | <0.001 |
| Aberrant Motor Behavior |  |  |  |  |  |  |  |
| log(HR) | 0.03 | -0.12 | -0.01 | 0.09 | 2.30 | 0.01 | 0.74 |
| 95% CI | -0.02, 0.07 | -0.75, 0.50 | -0.12, 0.10 | -0.13, 0.32 | 1.00, 3.60 | 0.00, 0.02 | 0.12, 1.40 |
| p-value | 1.000 | 1.000 | 1.000 | 1.000 | 0.005 | 0.595 | 0.243 |
| Sleep Disorders |  |  |  |  |  |  |  |
| log(HR) | -0.02 | 0.29 | 0.02 | -0.10 | 0.35 | 0.00 | -0.12 |
| 95% CI | -0.04, 0.00 | -0.04, 0.63 | -0.03, 0.08 | -0.20, -0.01 | -0.42, 1.10 | -0.01, 0.01 | -0.51, 0.26 |
| p-value | 1.000 | 0.990 | 1.000 | 0.466 | 1.000 | 1.000 | 1.000 |
| Appetite and Eating Disorders |  |  |  |  |  |  |  |
| log(HR) | 0.02 | -0.47 | 0.06 | -0.16 | 1.10 | 0.00 | 0.07 |
| 95% CI | -0.01, 0.04 | -0.83, -0.10 | -0.01, 0.13 | -0.27, -0.04 | 0.29, 2.00 | 0.00, 0.01 | -0.37, 0.51 |
| p-value | 1.000 | 0.152 | 0.863 | 0.078 | 0.098 | 1.000 | 1.000 |

# Supplementary Figure


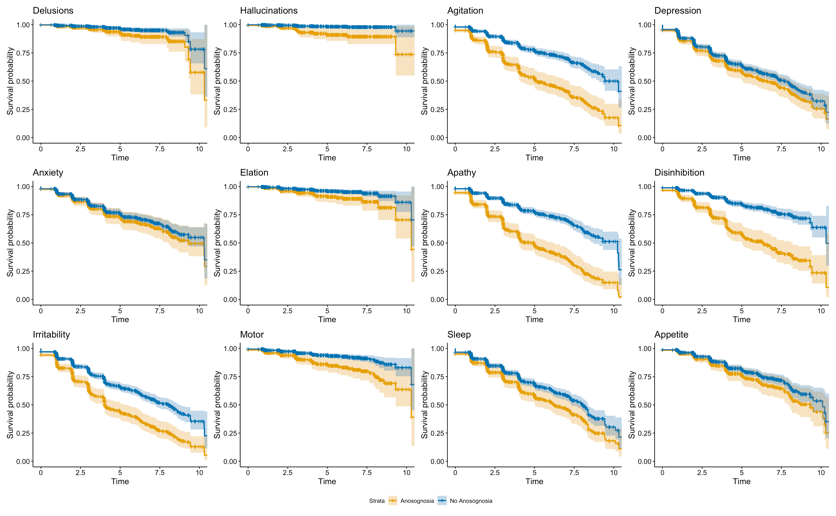


**Supplementary Figure 1.** Cox regression model outputs comparing the onset of NPS over time for participants with and without stable anosognosia. The orange line indicates participants with anosognosia, and the blue line indicates participants without anosognosia.
